# Structural Alterations in a Rat Model of Short-Term Conductive Hearing Loss Are Associated With Reduced Resting State Functional Connectivity

**DOI:** 10.3389/fnsys.2021.655172

**Published:** 2021-08-12

**Authors:** Francis A. M. Manno, Ziqi An, Rachit Kumar, Ed X. Wu, Jufang He, Yanqiu Feng, Condon Lau

**Affiliations:** ^1^Department of Physics, City University of Hong Kong, Hong Kong, SAR China; ^2^Guangdong Provincial Key Laboratory of Medical Image Processing, School of Biomedical Engineering, Southern Medical University, Guangzhou, China; ^3^Key Laboratory of Mental Health of the Ministry of Education, Guangdong-Hong Kong-Macao Greater Bay Area Center for Brain Science and Brain-Inspired Intelligence, Southern Medical University, Guangzhou, China; ^4^Perelman School of Medicine, University of Pennsylvania, Philadelphia, PA, United States; ^5^Medical Scientist Training Program, University of Pennsylvania, Philadelphia, PA, United States; ^6^Department of Electrical and Electronic Engineering, The University of Hong Kong, Hong Kong, SAR China; ^7^Laboratory of Biomedical Imaging and Signal Processing, The University of Hong Kong, Hong Kong, SAR China; ^8^Department of Neuroscience, City University of Hong Kong, Hong Kong, SAR China; ^9^Department of Biomedical Sciences, City University of Hong Kong, Hong Kong, SAR China

**Keywords:** conductive hearing loss, resting state functional connectivity, structural MRI, diffusion tensor imaging, rat – brain

## Abstract

Conductive hearing loss (CHL) results in attenuation of air conducted sound reaching the inner ear. How a change in air conducted sound alters the auditory system resulting in cortical alterations is not well understood. Here, we have assessed structural and functional magnetic resonance imaging (MRI) in an adult (P60) rat model of short-term conductive hearing loss (1 week). Diffusion tensor imaging (DTI) revealed fractional anisotropy (FA) and axial diffusivity alterations after hearing loss that circumscribed the auditory cortex (AC). Tractography found the lateral lemniscus tract leading to the bilateral inferior colliculus (IC) was reduced. For baseline comparison, DTI and tractography alterations were not found for the somatosensory cortex. To determine functional connectivity changes due to hearing loss, seed-based analysis (SBA) and independent component analysis (ICA) were performed. Short term conductive hearing loss altered functional connectivity in the AC and IC, but not the somatosensory cortex. The results present an exploratory neuroimaging assessment of structural alterations coupled to a change in functional connectivity after conductive hearing loss. The results and implications for humans consist of structural-functional brain alterations following short term hearing loss in adults.

## Introduction

### Impact of Conductive Hearing Loss

Conductive hearing loss (CHL) results in the attenuation of air conducted sound (Willcox and Artz, 2007). Bilateral CHL produces air-conducted sound attenuation but does not raise bone-conducted thresholds (indicating no cochlear damage: Tucci et al., 1999). The loss of peripheral sound input (i.e., afferent) results in a dramatic decrease to inhibitory synaptic strength (GABA is decreased; Kotak et al., 2008, 2013) and increase in excitatory strength (Glutamate is increased – Kotak and Sanes, 1997; Kotak et al., 2005) in the lateral superior olive auditory pathway. Interestingly, the emergence of the inhibitory synaptic strength results in a critical period where deficits can be induced in juveniles, but not adults (Takesian et al., 2012). Early CHL induced by malleus removal results in task-specific behavioral threshold increases of ≈35 decibel (dB) for sinusoidally amplitude modulated (AM) stimuli and increases of ≈40 dB in neural auditory brainstem responses to 100 μs clicks or 4 ms pure tones (Rosen et al., 2012). Additionally, developmental CHL animals display slower rates of acquisition for AM discrimination tasks due to an impaired ability to generalize newly introduced stimuli (von Trapp et al., 2017). The perceptual deficits found in CHL are likely due to aberrant processing in the auditory cortex (AC; i.e., decrease of inhibitory synaptic strength; Yao and Sanes, 2018, argue not in the auditory brainstem). Nevertheless, efferent pathways are also altered by CHL (Liberman et al., 2015). Here it was shown that the density of olivocochlear terminals in the cochlear epithelium is reduced (Liberman et al., 2015). We recognize the variety of studies in CHL complicate any general conclusions, as the majority of studies were conducted during development. The auditory system is greatly impacted due to CHL, yet investigations into structural-functional correlations in hearing loss are lacking, with studies concentrating either on structural or functional approaches.

### Animal Models of Hearing Loss Using Magnetic Resonance Imaging

We have recently assessed the structural foundations of hearing loss across the lifespan in humans using a meta-analysis and meta-regression (Manno et al., 2021). In humans, we found that hearing loss not only affects auditory structures, but is brain-wide, multi-focal, and impacts regions and tracts differently depending on auditory input and compensatory mechanisms. In rats, we have demonstrated acute and chronic noise exposure result in structural and functional changes in auditory structures from the midbrain to the cortex (Abdoli et al., 2016; Yang et al., 2018; Wong et al., 2020). In long-term passive noise exposure, diffusion tensor imaging (DTI), voxel-based statistics (VBS) revealed greater fractional anisotropy (FA) in the pyramidal tract and decreased FA in the tectospinal tract and trigeminothalamic tract (Abdoli et al., 2016). Acute noise exposure showed a reduction in midbrain sound-evoked responses as early as 1 week, as assessed using functional magnetic resonance imaging (fMRI) (Yang et al., 2018). Using a salicylate-induced model of hyperacusis, we found AC tone-evoked fMRI signals to 80 decibel sound pressure level (dB SPL) with 8, 16, and 32 kHz stimuli during salicylate treatment were consistently larger than saline (indicating evidence of enhanced central gain; Wong et al., 2020). In contrast, the lateral lemniscus and inferior colliculus (IC) fMRI response obtained during salicylate treatment were similar to saline control values (Wong et al., 2020). Rodent models of hearing loss provide valuable information for establishing the basis of pathophysiology (Salvi et al., 2020) and further investigations should study the variety of hearing losses which occur in humans.

### Present Study Questions

In the present experiments we sought to explore the structural-functional correlations of a rat model of CHL using neuroimaging techniques. Here, we used DTI and fMRI to determine how CHL alters the rat brain over a short duration. The present study was a repeated analysis self-controlled design, scanning rats prior to and 1-week after CHL induction. We ask the following questions: (1) what structural alterations can be revealed by DTI after CHL induction and (2) do these structural alterations result in functional deficits? The present study found reduced DTI metrics in the AC, reduced tract length and density of the lateral lemniscus as assessed by DTI, and reduced resting-state fMRI (rsfMRI) signal functional connectivity in the AC. Future studies should examine structural-functional relationships long term and therapeutic regimens to rehabilitate hearing loss models.

## Materials and Methods

### Animals Preparation

Animals were prepared for fMRI experiments as described in our earlier studies (Lau et al., 2011, 2013, 2018; Cheung et al., 2012a,b; Lau et al., 2015a,b; Wong et al., 2017; Dong et al., 2018; Manno et al., 2019). All aspects of this study were approved by the Committee on the Use of Live Animals in Teaching and Research at the City University of Hong Kong. The present experiments consisted of behavioral assays and structural-functional MRI. For structural-functional MRI experiments, male Sprague-Dawley rats (*n* = 12; 250 g, postnatal day 60) were scanned before and 1-week after CHL induction (repeated analysis self-control design). Rodents were cage-housed under a constant 25°C temperature and 60–70% humidity. After surgery, the animals were returned to their home cages under warm conditions for recovery, and they were housed under a 12:12-h light/dark cycle in a temperature-controlled room with *ad libitum* access to food and water. Rodents were acclimated to the housing environment for at least one day prior to the experiment. Anti-inflammatory drugs were supplied in the water for 1 week. The experiments were conducted in a repeated analysis design where CHL was induced after an initial control period, and animals were subsequently used as their self-control for all experiments.

### Conductive Hearing Loss Procedure

**FIGURE 1:**
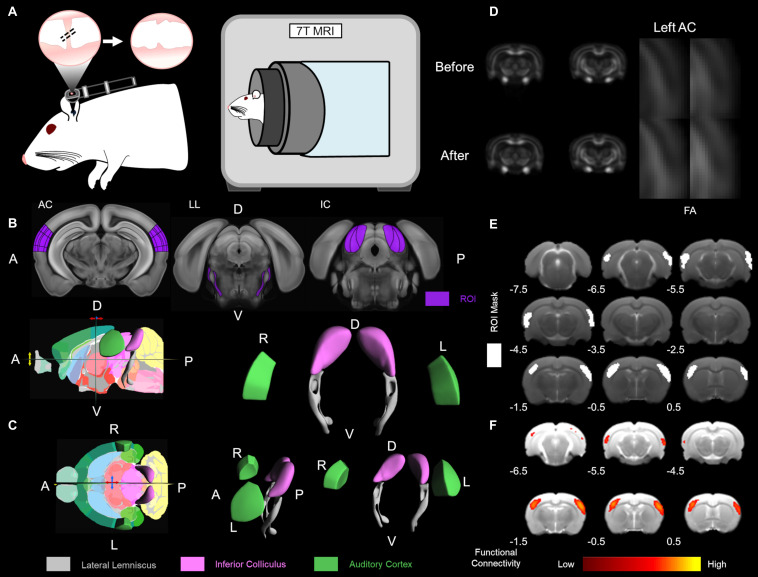
Experimental design and data analysis. **(A)** The CHL surgery was conducted by tympanic membrane (TM) puncture and malleus displacement. A schematic of the rat ear with the otoscope highlighting the TM is depicted. Hash marks across the malleus indicate displacement and TM puncture with the serrated TM on the right. To the immediate right, the 7 Tesla magnetic resonance imaging (MRI) scanner for rodent structure-functional MRI (PharmaScan, Bruker Biospin). The Allen Mouse Brain Reference Atlas was used for visualization (Lein et al., 2007). **(B)** The Allen Mouse Brain Reference Atlas average template (Lein et al., 2007) with the region of interest (ROI), in dark purple, from left to right: AC 80 image, LL 96 image, and IC 101 image. **(C)** The Allen Mouse Brain Atlas 3D rendering a cutaway of the longitudinal/sagittal plane (top) and horizontal/transverse plane (bottom) with the 2D coronal reference atlas (Lein et al., 2007). The Allen Mouse Brain Atlas 3D volumetric rendering tilted in the horizontal plane rotated left 5° (top), tilted in the horizontal plane rotated left 20° (bottom), and tilted in the horizontal plane rotated left 90° (bottom left), all in the y-axis translation. Volumetric ROI are color-coded lateral lemniscus in gray, inferior colliculus (IC) in light purple (lavender) and auditory cortex in green. R – right, L – left, D – dorsal, V – ventral, A – anterior, P – posterior. **(D)** Diffusion tensor imaging (DTI) fractional anisotropy (FA) of the before and 1 week after CHL timepoints. The left auditory cortex (AC) is highlighted in the zoomed insert. **(E)** The ROI for masks used for functional connectivity are delineated in white. Bregma coordinates (in mm) are below the brain. **(F)** Functional connectivity maps using a color bar depicted from the correlation coefficient and z-score.

The CHL induction was a modified procedure from Tucci et al. (1999) (Figure 1A; Manno et al., 2020). Rats were anaesthetized with a cocktail of ketamine and xylazine (80–100 mg/kg: 5–10 mg/kg) via intraperitoneal injection. A surgical field was setup and the head of the rat was aligned according to the prone position, closest to the experimenter. The helix of the rat ear was extended to cause the external auditory canal (i.e., ear canal and external auditory meatus) to become perpendicular to the surface of the tympanic membrane (TM). Micro-scissors (Micro spring scissors, RWD Life Science, and S11035-08) were introduced in the center of the auditory canal paying attention not to skim/nick the skin of the canal (Figure 1A). The scissors were introduced slowly, approximately 5 mm depth from the center of obscurity. The scissors were thrusted forward gently through the center of the TM. The thrusting elicited TM puncture (Figure 1A). With the micro-scissors in the appropriate position and correct depth, a “pop” sound was noticed when the micro-scissor tips puncture the TM displacing the malleus (Figure 1A). The popping sound was ≈20 dB SPL from background sound change as recorded by a high frequency microphone (M50, Earthworks, Chesterfield, MO, United States). After puncturing the TM, the micro-scissors were immediately opened and rotated three times to ensure displacement of the head of the malleus (Figure 1A). The micro-scissors were removed, and the rat placed under otoscope to visualize and ensure TM puncture (Otoscope mini 3000, HEINE, D-008.70.120M, and Standard LED otoscope). It is important to note no significant bleeding after the surgical procedure. Each rat was evaluated before and after CHL underneath otoscope for visualization and confirmation of normal TM and damaged TM after CHL induction. After CHL induction, the head of the malleus should be missing and not visible through the TM (Figure 1A). Anti-inflammatory drugs were supplied in the water for 1 week.

### Magnetic Resonance Imaging Acquisition

Experiments were performed on a seven Tesla MRI scanner with a maximum gradient of 360 mT/m (70/16 PharmaScan, Bruker Biospin, Ettlingen, and Germany) using a transmit-only birdcage coil in combination with an actively decoupled receive-only surface coil (Figure 1A; Manno et al., 2019). The animals were initially anesthetized with 3% isoflurane. When sufficiently anesthetized, 1–2 drops of 2% lidocaine were applied to the cords to provide local anesthesia before the endotracheal intubation. The animals were mechanically ventilated at a rate of 54–56 min^–1^ with 1–1.5% isoflurane in room-temperature air using a ventilator (TOPO, Kent Scientific Corp., Torrington, CT, United States). During MRI, the animals were placed on a plastic cradle with the head fixed with a tooth bar and plastic screws in the ear canals. Rectal temperature was maintained at ∼37.0°C using a water circulation system. Continuous physiological monitoring was performed using an MRI-compatible system (SA Instruments, Stony Brook, NY, United States). Vital signs were within normal physiological ranges (rectal temperature: 36.5–37.5°C, heart rate: 350–420 beat/min, breathing: 54–56 breath/min, and oxygen saturation: N95%) throughout the duration of the experiment (Chan et al., 2009; Lau et al., 2011; Cheung et al., 2012a; Zhou et al., 2014). Scout T2-weighted (T2W) images were first acquired to position the subsequent images in a reproducible manner. The scan geometry was four 1.0 mm thick slices along the coronal view, with 0.1 mm interslice distance, positioned according to the rat brain atlas (Paxinos and Watson, 2004) such that the third slice was centered on the IC (Bregma −8.6 mm; Figure 1B).

### Diffusion Tensor Imaging Acquisition

The DTI scan sequence was a spin-echo 4-shot echo planar imaging sequence with 30 diffusion gradient directions and *b*-value = 1,000 s/mm^2^, and five images without diffusion sensitization (*b* = 0.0 ms/μm^2^, b0 images) were acquired. Images with motion artifacts were discarded. The imaging parameters were: repetition time (TR)/echo time (TE) = 3,000/31.6 ms, δ/Δ = 5/17 ms, field of view = 3.2 × 3.2 cm^2^, data matrix = 128 × 128, up-sampled data matrix = 256 × 256, cropped data matrix = 128 × 128, and number of excitements = 4. Anatomical images were acquired with rapid acquisition with refocusing echoes (RARE). The imaging parameters were RARE factor = 8, TR/TE = 4,200/32 ms, field of view = 3.2 × 3.2 cm^2^, data matrix = 256 × 256.

### Diffusion Tensor Imaging Analysis

The diffusion-weighted images and b0 images were realigned and normalized to the b0 image of a template rat using SPM12 (Wellcome Trust Centre, Oxford, United Kingdom) and custom Matlab scripts (The MathWorks, Natick, MA, United States). DTI index maps were calculated by fitting the diffusion tensor model to the diffusion data at each voxel using DTIStudio v3.02 (Chan et al., 2009; Hui et al., 2010).

A map of color-coded directionality was generated, in which the color codes were for the principal eigenvector orientation while the contrast was weighted with FA. The following was used for depiction, white matter tracts running in the X-, Y-, and Z- directions were coded red, green, and blue, respectively (Pajevic and Pierpaoli, 1999). Therefore, the major eigenvector directional components for color-coded tractography (cFA) used the following naming convention: blue for superior-inferior, red for left-right, and green for anterior-posterior orientations (Jellison et al., 2004; Zhang et al., 2006). A voxel-wise *t*-test was performed on each DTI indices maps between the control and CHL rats to generate VBS. DTI images were spatially normalized by non-linear registration to the T2 structural image of the same rat. In brief, b0 and the T2 image were co-registered by a rigid transformation, followed by a two-dimensional affine transformation and a non-linear registration to improve the mapping between DTI and the structural image. The DTI index maps were calculated by fitting the diffusion tensor model to the diffusion data at each voxel using DTIStudio v3.02 (Johns Hopkins University, Baltimore, MD, and United States) as previously detailed (Chan et al., 2009; Hui et al., 2010; Abdoli et al., 2016). The normalized maps were smoothed with a 0.3 mm Gaussian kernel. The first and last slices were excluded in order to avoid truncation artifacts. The VBS clusters were considered significant at threshold *p* < 0.05 and cluster size > 3 voxels. The structures indicated by clusters were identified using the rat brain atlas (Paxinos and Watson, 2004; Figures 1B,C).

The region of interest (ROI) analysis (Figure 1B) was used to confirm that the structures identified by VBS were not significantly affected by normalization, registration, or other processing errors. Due to its confirmatory purpose, the ROI analysis was done in a conservative manner on unnormalized images. For each VBS identified structure, left and right hemisphere ROIs were drawn (Figure 1C). The ROIs were drawn such that they would be exclusive to the identified structure regardless of which rat they were applied to resulting in ROIs that were slightly smaller than the actual structure but still representative of the structure’s general shape and size. All ROIs were of identical shape, size, and the left and right ROIs were always placed along the same horizontal axis. The only variation between the subjects was the distance between the right and left ROI. The atlas was used to determine anatomical landmarks, such as the edge of the brain as the starting point of the lateral lemniscus tract, to serve as guidelines for placement of the ROIs. Once the ROIs were placed, FA, MD, RD (λ_⊥_), and AD (λ_//_) values were recorded for the left and right structures (Figure 1D). Values were recorded from the left and right sides to ensure that changes were occurring bilaterally.

Following ROI analysis, tractography was performed to ensure that ROIs drawn corresponded to the intended white matter structures. The ROI for the lateral lemniscus was placed on Bregma −7.3, −8.3, and −9.3 mm and used to generate tracks. Tracking was performed on a voxel-by-voxel basis by continuously following the orientation of the first eigenvector of the tensor. All fiber bundles passing through the ROI on the second and third coronal slice which had a minimum FA threshold of 0.3 were followed until their FA was under 0.2 or the turning angle was greater than 60°. Using this methodology, the ventral aspect of the lateral lemniscus was delineated. The lateral lemniscus connects the IC to the lower brainstem superior olivary complex to the cochlear nucleus (Winer and Schreiner, 2005). The lateral lemniscus is a prominent pathway in GABAergic inhibition to the central nucleus of the IC (Li and Kelly, 1992; Zhang et al., 1998).

### Functional Magnetic Resonance Imaging Acquisition

Similar to DTI, an anatomical image was first acquired using a 2D RARE sequence. The sequence parameters were RARE factor = 8, field of view = 32 × 32 mm^2^, data matrix = 256 × 256, TR = 4200 ms, and TE = 36 ms. fMRI images were acquired with a gradient echo echo planar imaging (GE-EPI) sequence with the following parameters: field of view = 32 × 32 mm^2^, data matrix = 64 × 64, TR = 1,000 ms, TE = 20 ms, and 400 acquisitions. The EPI scan geometry was imported from the anatomical scan geometry. Twelve EPI scans were performed.

### Reduced Resting-State fMRI Analyses

For each rsfMRI session, all images were first corrected for slice timing differences and then realigned to the first image of the series using SPM12 (Wellcome Department of Imaging Neuroscience, University College, London). A voxel-wise linear detrending with least-squares estimation was performed temporally to eliminate the baseline drift caused by physiological noise and system instability. No spatial smoothing was performed while a temporal band-pass filtering (0.005–0.1 Hz) was applied. The first 15 image volumes and last 15 image volumes of each session were discarded to eliminate possible non-equilibrium effects. Finally, high-resolution anatomical images from individual animals were coregistered to a custom-made brain template acquired from a separate age-matched rat with the same settings with a 3D rigid-body transformation and the transforming matrix was then applied to the respective rsfMRI data (Chan et al., 2015, 2017). To determine functional connectivity changes before and after CHL, seed-based analysis (SBA) and independent component analysis (ICA) were performed.

For SBA, two 3 × 3 voxel regions were chosen as the ipsilateral and contralateral seed, respectively, in the primary AC, primary somatosensory cortex (S1) and the IC. Regionally averaged time course from the voxels within each seed served as the respective reference time course. Pearson’s correlation coefficients were calculated between the reference time course and the time course of every other voxel to generate two rsfMRI connectivity maps for each region. A 3 × 3-voxel region on the contralateral side of the seed was defined as the ROI (Figure 1E). Interhemispheric functional connectivity for each region was then quantified by averaging the correlation coefficient value of the corresponding ipsilateral and contralateral ROI (Chan et al., 2015, 2017; Figure 1F).

For ICA, coregistered rsfMRI data was analyzed with the GIFT v4.0b Toolbox. The Infomax algorithm was used and group-level ICA was performed on all rsfMRI data at the same time point. The group-level spatial ICA maps of independent resting-state networks were scaled to z-scores with a threshold of *z* > 2.5 (corresponding to a significance level of *p* < 0.01). The ICA maps were then visually inspected and labeled based on the spatial patterns in reference to known anatomical and functional locations. AC and S1 resting-state networks were further quantified. ROIs covering the bilateral functional areas were defined according to the resting-state networks obtained (Figure 1E). Mean z-scores were obtained by averaging the values within the ROIs (Chan et al., 2015; Figure 1F).

## Results

### Structural Alterations Underlying CHL Assessed by VBS of DTI Images (Figure 2)

**FIGURE 2:**
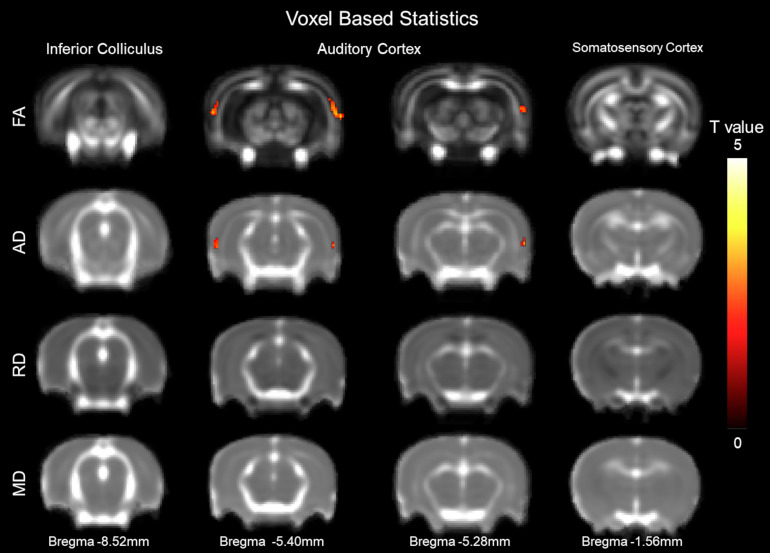
Structural alterations underlying conductive hearing loss assessed by DTI voxel-based statistics. The y-axis representing rows are fractional anisotropy (FA), axial diffusivity (AD, λ//), radial diffusivity (RD, λ_⊥_), and mean diffusivity (MD). Columns represent different Bregma locations –8.6, –6.40, –5.3, and –2.0 mm. Color bars represent positive t value differences between before and after CHL (warm colors from 0 to 5). The maximum point of significance for FA was *t* = 3.170, and for AD was *t* = 2.729.

Voxel-based statistics were used to visualize areas altered 1 week after CHL (Figure 2). The VBS was mapped as a function of *t*-value change. The maximum point of significance for FA was *t* = 3.170 and for AD was *t* = 2.729. Clusters of voxels with significant differences were observed bilaterally in the AC. The visualization was done for Bregma −6.40 and −5.3 mm. The VBS analysis was followed by a DTI ROI analysis on the AC for determining FA, MD, RD (λ_⊥_), and AD (λ_//_).

### Structural Alterations Underlying CHL Assessed by DTI ROI Analysis (Figure 3)

**FIGURE 3:**
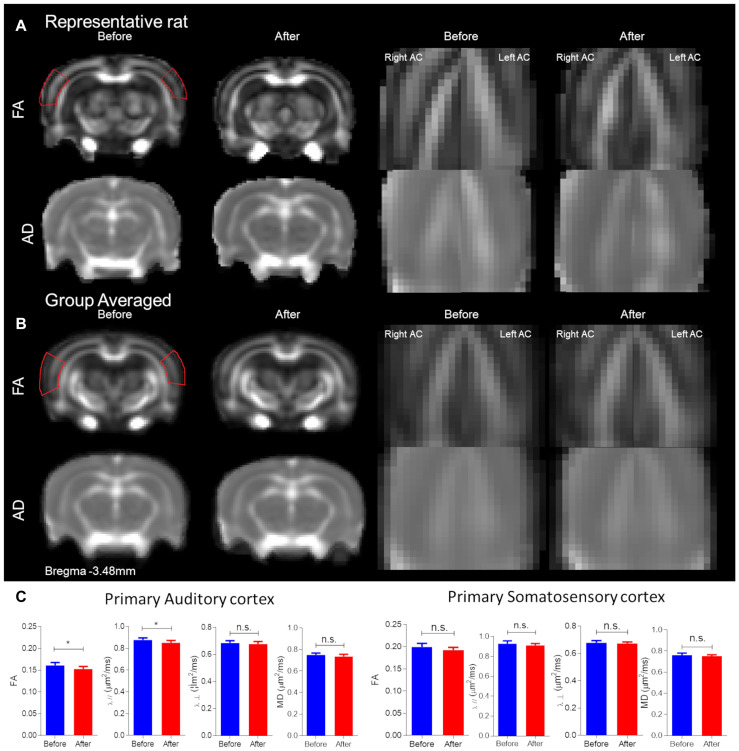
Structural alterations underlying conductive hearing loss assessed by DTI ROIs. The panel is segregated into before and after CHL for Bregma –3.48 mm. **(A)** Representative rat FA maps (first row of brains from left) and AD maps (second row of brains from left) from individual CHL rats. **(B)** Group-averaged FA (first row of brains from left) and AD (second row of brains from left) maps for CHL. Note the red outline for FA represents the approximate ROI for measures. **(C)** Bar plots of DTI measures for the primary auditory cortex and somatosensory cortex: FA, MD, RD (λ_⊥_), and AD (λ_//_) for before (blue) and after (red) CHL with standard deviation. The mean FA and AD after CHL were significantly decreased compared to before, *T*-test: *t* = 2.769, df = 12, *p* = 0.0143 and *t* = 2.474, df = 15, and *p* = 0.0258, respectively. The asterisk (*) indicates significant *p* < 0.05.

The primary AC was delineated, first by each individual rat, and then group-averaged (Figures 3A,B). The panel is segregated into before and after CHL for the left and right hemispheres on the coronal section Bregma −3.48 mm. The FA and AD for the primary AC was significantly decreased after CHL (Figure 3C). The mean FA for AC after CHL was significantly decreased compared to before (before FA 0.1612 ± 0.0020 and after FA 0.1526 ± 0.0021; *T*-test: *t* = 2.769, df = 12, *p* = 0.0143). The mean AD for AC after CHL was significantly decreased compared to before (before AD 0.8760 ± 0.0064 and after AD 0.8513 ± 0.0077; *T*-test: *t* = 2.474, df = 15, and *p* = 0.0258). No change was observed for the primary somatosensory cortex.

### Structural Alterations Underlying CHL Assessed by Tractography (Figure 4)

**FIGURE 4:**
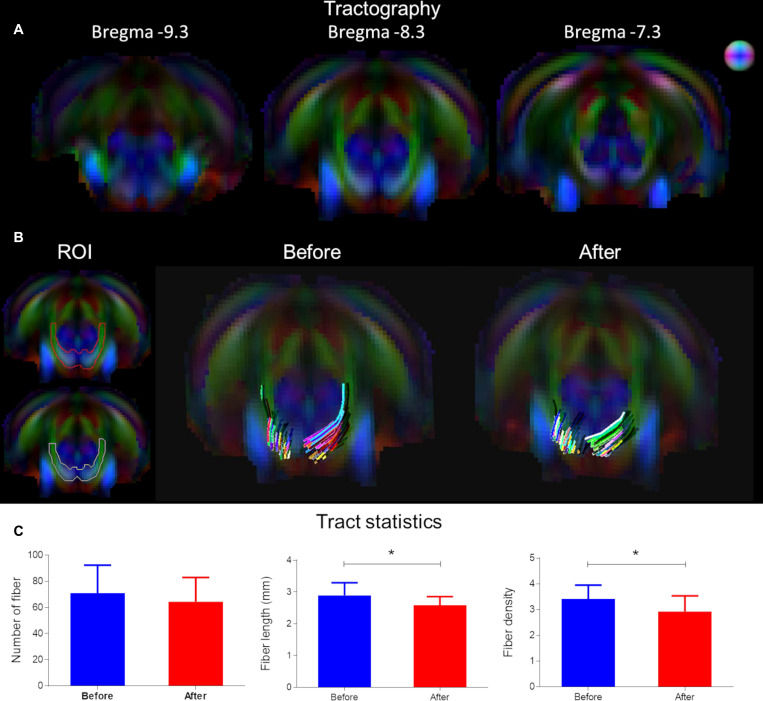
Tract alterations underlying conductive hearing loss assessed by tractography. **(A)** Tractography was used to delineate the ventral aspect of lateral lemniscus on Bregma –7.5, –8.6, and –9.7 mm. The color map shows the primary eigenvector in the region of the ventral lateral lemniscus traversing upward in the ventral-dorsal direction. **(B)** The tractography panel ROI demonstrates the fibers originate from a ventral position and course upward dorsally in a bilaterally symmetrical fashion. **(C)** Bar plots of tract metrics in the ROI for the lateral lemniscus. The mean fiber number was insignificantly different after CHL; however, the mean fiber length and mean fiber density for the lateral lemniscus after CHL was significantly decreased compared to before (*t*-test: *t* = 2.548, df = 30, *p* = 0.0162 and *t* = 2.388, df = 30, and *p* = 0.0234, respectively).

Tractography was used to delineate the ventral aspect of lateral lemniscus, a prominent tract in the rat (Winer and Schreiner, 2005; Figure 4A). The color map shows the primary eigenvector in the region of the ventral aspect of the lateral lemniscus is along the posterior–anterior direction and traversing upward in the ventral-dorsal direction (Figure 4B – ROI). The tractography panel (Figure 4B) demonstrates the fibers originate from a ventral position and course upward dorsally in a bilaterally symmetrical fashion. Analysis of fiber number, fiber length and fiber density using tractography found CHL elicited a significant reduction in tract length and fiber density (*p* < 0.05; Figure 4C). The mean fiber number for the lateral lemniscus after CHL was insignificantly different for CHL (before 71.07 ± 5.684, *N* = 14; after 64.39 ± 4.381, *N* = 18; *T*- test: *t* = 0.9474, df = 30, and *p* = 0.3510). The mean fiber length for the lateral lemniscus after CHL was significantly decreased compared to before (before 2.891 mm ± 0.1076 mm, *N* = 14; after 2.590 mm ± 0.06218 mm, *N* = 18; *T*-test: *t* = 2.548, df = 30, and *p* = 0.0162). The mean fiber density for the lateral lemniscus after CHL was significantly decreased compared to before (before 3.414 mm^3^ ± 0.1422 mm^3^, *N* = 14; after 2.923 mm^3^ ± 0.1438 mm^3^, *N* = 18; *T*-test: *t* = 2.388, df = 30, and *p* = 0.0234).

### Functional Connectivity Alterations After CHL as Assessed by rsfMRI (Figure 5)

**FIGURE 5:**
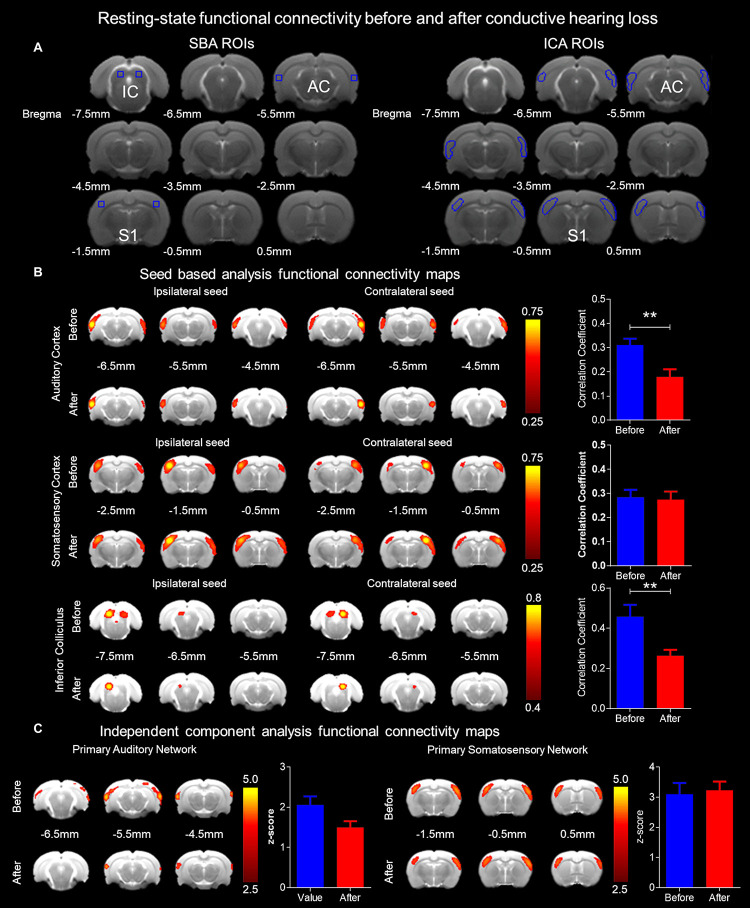
Functional connectivity alterations resulting from conductive hearing loss as assessed by rsfMRI. **(A)** Seeds used for SBA and masks for ICA were used to delineate ROI for the bilateral auditory cortex, somatosensory cortex, and IC rsfMRI networks. **(B)** The SBA rsfMRI connectivity was significantly decreased in the auditory cortex and IC after CHL (*p* < 0.01), however, remaining unchanged in the somatosensory cortex. The bar plots indicate the correlation coefficient before (blue) and after (red) CHL, demonstrating a decrease of 42.27% and 42.50% for the auditory cortex and IC, respectively. **(C)** The ICA rsfMRI connectivity was decreased in the auditory cortex after CHL, and was unchanged in the somatosensory cortex. The bar plots indicate the z-score before (blue) and after (red) CHL, demonstrating a decrease of 27.11% for the auditory cortex. Both SBA and ICA demonstrate rsfMRI networks decrease after CHL.

Figure 5A shows ROIs delineating the seeds from SBA and the masks from ICA. The mean correlation coefficient maps (Figure 5) demonstrate the presence of the bilateral auditory and somatosensory cortices, and IC rsfMRI networks before and after CHL. For rsfMRI, 8–10 rats passed the quality control for SBA (df = 20) and ICA (df = 18) assessment. The SBA correlation coefficient for rsfMRI connectivity was significantly decreased in the AC by 42.27% (Bonferroni’s *post hoc* test, *p* < 0.01) after CHL (before 0.3106 ± 0.9178 CI: 0.2523–0.3689 and after 0.1793 ± 0.1094 CI: 0.1098–0.2488, *t* = 3.185, df = 22, and *p* = 0.0043). Note that the bilateral somatosensory network revealed no significant connectivity strength changes between the before and after CHL timepoint (before: 0.2728 ± 0.0890; CI: 0.2091–0.3365, and after: 0.2749 ± 0.1043; CI: 0.2003–0.3495, *t* = 0.04890, df = 18, *p* = 0.9615, difference between means: 0.21 ± 4.347). The SBA correlation coefficient for rsfMRI connectivity was significantly decreased in the IC by 42.50% (Bonferroni’s *post hoc* test, *p* < 0.01) after CHL (before: 0.4576 ± 0.1867 CI: 0.3241–0.5912 and after: 0.2631 ± 0.09272 CI: 0.1967–0.3294, *t* = 2.952, df = 18, and *p* = 0.0085). The SBA revealed a bilateral decrease in rsfMRI connectivity in the AC and IC after CHL (Figure 5B) indicating these ROI became less functionally connected.

The z-score maps (Figure 5C) demonstrate the presence of the bilateral auditory and somatosensory cortex rsfMRI networks before and after CHL. The ICA z-score for rsfMRI connectivity was decreased in the AC by 27.11% after CHL (before: 2.058 ± 0.5206 CI: 1.512–2.605 and after: 1.500 ± 0.3815 CI: 1.100–1.901) *t* = 2.117, df = 10, and *p* = 0.063. Note that bilateral somatosensory network revealed no significant connectivity strength changes between the before and after CHL timepoint (before: 3.155 ± 0.6749 CI: 2.727–3.584 and after: 3.037 ± 0.7700 CI: 2.547–3.526) *t* = 0.4020, df = 22, *p* = 0.6915, difference between means: 11.88 ± 29.56). The ICA revealed a bilateral decrease in rsfMRI connectivity in the AC after CHL (Figure 5C), indicating the ROI became less functionally connected.

## Discussion

Bilateral CHL produces air-conducted sound attenuation (indicating no cochlear damage: Tucci et al., 1999), resulting in task-specific behavioral threshold increases of ≈35 dB and neural auditory brainstem response threshold increases of ≈40 dB for sinusoidally AM stimuli (Rosen et al., 2012). The auditory system is greatly impacted due to CHL, yet investigations into structural-functional correlations in hearing loss are lacking. Here we demonstrated CHL results in structural and functional alterations in the IC and AC. Structurally, FA and axial diffusivity alterations are found in the AC and tractography revealed the lateral lemniscus tract leading to the inferior cortex bilaterally was reduced. Functionally after CHL, SBA revealed functional connectivity decreased in the AC and IC, while ICA revealed functional connectivity decreased in the AC, while both these assessments found no change in functional connectivity in the somatosensory cortex. The present results indicate structural alterations from short term hearing loss are associated with altered functional connectivity.

Rodent models recapitulate some of the main features of the neuropathology of human hearing loss. The research in rats and mice range from brief noise exposure to chronic conditions. In rats, long-term passive noise exposure has revealed greater FA in the pyramidal tract and decreased FA in the tectospinal tract and trigeminothalamic tract (Abdoli et al., 2016). These tracts are involved in voluntary control of the body and limbs, transmit noxious stimuli from the face and motor pathway which reflexively controls the muscles of the head for visual stimuli, for the pyramidal, trigeminothalamic, and tectospinal tracts, respectively (Abdoli et al., 2016). In the rat, these tracts possibly help to orient the rat to an alarm such as the acoustic startle reflex; thus, adjusting body coordination to sound stimuli. This is interesting as a previous experiment has demonstrated reflex inhibition produced by brief noise exposure (Cory and Ison, 1979; Wu et al., 1984), indicating in the rat a priming or sensitization effect. It is interesting to note, an increased sensitivity to continuous noise results in suppression in the acoustic startle response (ASR) in rats (Rybalko et al., 2019). In the present study, we demonstrated an approximate ≈35 dB attenuation (Sohmer et al., 1991; Sohmer and Friedman, 1992; Rosen et al., 2012; Ye et al., 2021), resulted in a decrease in fiber length and fiber density in the lateral lemniscus and decrease in FA and AD for AC after 1-week of hearing loss in postnatal day 60 rats. The current study performed hearing loss at postnatal day 60 (considered young adults, McCutcheon and Marinelli, 2009) and examined changes over a brief period of time (1 week). A study measuring the impact of conductive hearing loss induced developmentally (postnatal day 10) found perceptual deficits later on in adulthood (postnatal day 70). Juvenile onset hearing loss (89 Hz) had greater frequency modulation detection thresholds (89 Hz) than adult onset hearing loss (64 Hz; Buran et al., 2014). The study indicated decreased auditory stimulation at an early age had shallower psychometric functions likely leading to altered brain development. In the current study, we demonstrate what the altered brain looks like due to impoverished hearing in adults, possibly more affected if induced during the development, altricial period. Interestingly, in salicylate- and noise-induced tinnitus model, the IC exhibited supernormal uptake manganese-enhanced MRI implicating its role in tinnitus-related neuronal activity (Holt et al., 2010). The IC is a prominent site of brain stem convergence and multisensory integration in the auditory pathway of the rat (Winer and Schreiner, 2005). The results support the conclusion that the IC (Figure 5C) and tracts leading to this midbrain structure such as the lateral lemniscus (Figure 4B) are intimately involved in the first processes of structural-functional reorganization due to CHL. Its likely the cochlear nucleus, lateral lemniscus and IC, in addition to other midbrain structures (e.g., superior olivary complex; Malmierca, 2003; Osen et al., 2019), are affected in a reentrant manner, first with lack of external stimuli (bottom-up) and second with the release of top-down processing (Caras and Sanes, 2017). Future experiments should attempt to distinguish the relative contributions to both processing pathways (top–down/bottom–up). Further, using a salicylate-induced model of hyperacusis, we found AC tone-evoked BOLD fMRI signals during salicylate treatment were consistently larger than saline (indicating evidence of enhanced central gain; Wong et al., 2020). Interestingly during this model of hyperacusis, the lateral lemniscus and IC BOLD response obtained during salicylate treatment were similar to saline control values (Wong et al., 2020), indicating a top-down first contribution. Acute noise paradigms have used fMRI and implanted electrodes to mixed results. Acute noise exposure reduced midbrain IC sound-evoked responses as detected using fMRI by as early as 1 week (Yang et al., 2018). Using implanted electrodes in an acoustic startle–reflex paradigm, a decrease in sound evoked potential was seen in IC; however, an increase was observed in AC, after noise exposure (Sun et al., 2012). In the present study with rats, we have demonstrated short-term early affect to auditory structures due to CHL. Future studies should establish the interrelations between these structures over the longitudinal course of hearing loss.

In humans, we have recently assessed the structural foundations of hearing loss across the lifespan (Manno et al., 2021). Our systematic review and meta-analysis revealed *n* = 72 studies with structural alterations measured by MRI (bilateral = 64, unilateral = 8). The studies contained more than 66,545 variable datapoint metrics categorizing hearing loss into congenital and acquired cases from mild to profound impact (*n* = 7445) and control cases (*n* = 2924). This large longitudinal study found the endophenotype of hearing loss is heterogeneous, determined by gray matter alterations, and results in a widespread impact to the brain of pediatric, adult and aged-adult populations whether the etiology is congenital or acquired. The most important finding of our study was hearing loss is a unique sensory disorder, resembling a diffuse brain disorder, impacting regions and tracts differently depending on auditory input and compensatory mechanisms. In humans, several fMRI studies have documented the functional effects to hearing loss (Schmidt et al., 2013; Liu et al., 2015; Puschmann and Thiel, 2017; Xia et al., 2017; Fitzhugh et al., 2019; Luan et al., 2019, 2020; Wang et al., 2019; Wolak et al., 2019; Xu et al., 2019), although primarily congenital bilateral hearing loss. Nevertheless, reduced rsfMRI connectivity has been found to accompany hearing loss (as would be expected to AC, when sound stimuli is reduced) based on tone stimulation paradigms (Scheffler et al., 1998; Propst et al., 2010; Tibbetts et al., 2011). The functional MRI studies complement our structural MRI study indicating changes to structural-functional coupled relationships can be attributed to widespread brain regions due to the impact of hearing loss.

There are several limitations in this assessment of CHL structure-functional coupling. The sample size in the current study is relatively low (*N* = 9–12, separated by 1-week), which may have contributed to the low signal to noise ratio in the rsfMRI data quality. Future studies should consider increasing the sample size to address data quality concerns as well as imaging multiple timepoints to develop a longitudinal assessment of CHL. Furthermore, our DTI processing pipeline (Manno et al., 2019) may not have been sensitive enough to detect all the alterations seen in CHL during this short-term period. Optimization and improvement in the pipeline and scanning protocol for subsequent CHL studies might allow more accurate detection of structural-functional alterations for a variety of structures impacted due to hearing loss.

## Data Availability Statement

The datasets presented in this study can be found in online repositories. The names of the repository/repositories and accession number(s) can be found below: https://francismanno.github.io/fmanno/.

## Ethics Statement

The animal study was reviewed and approved by the Animal Research Ethics Sub-Committee of the City University of Hong Kong.

## Author Contributions

CL, YF, and FM organized the CHL project at The City University of Hong Kong. FM, ZA, RK, YF, and CL designed research. FM, ZA, and YX performed the research and data curation. FM, ZA, and RK analyzed the data. FM, ZA, RK, EW, JH, YF, and CL wrote and edited the manuscript. YF and CL acquired funding.

## Conflict of Interest

The authors declare that the research was conducted in the absence of any commercial or financial relationships that could be construed as a potential conflict of interest.

## Publisher’s Note

All claims expressed in this article are solely those of the authors and do not necessarily represent those of their affiliated organizations, or those of the publisher, the editors and the reviewers. Any product that may be evaluated in this article, or claim that may be made by its manufacturer, is not guaranteed or endorsed by the publisher.
